# Efficacy and safety of SGLT2 inhibitors in heart failure: systematic review and meta‐analysis

**DOI:** 10.1002/ehf2.13169

**Published:** 2020-12-22

**Authors:** Javed Butler, Muhammad Shariq Usman, Muhammad Shahzeb Khan, Stephen J. Greene, Tim Friede, Muthiah Vaduganathan, Gerasimos Filippatos, Andrew J. Stewart Coats, Stefan D. Anker

**Affiliations:** ^1^ Department of Medicine University of Mississippi Medical Center 2500 N. State Street Jackson MS 39216 USA; ^2^ Department of Medicine Dow University of Health Sciences Karachi Pakistan; ^3^ Division of Cardiology Duke University Medical Center Durham NC USA; ^4^ Department of Medical Statistics University Medical Center Goettingen Goettingen Germany; ^5^ DZHK (German Center of Cardiovascular Research), partner site Goettingen Goettingen Germany; ^6^ Heart and Vascular Center Brigham and Women's Hospital Boston MA USA; ^7^ National and Kapodistrian University of Athens School of Medicine Athens University Hospital Attikon Athens Greece; ^8^ Department of Cardiology IRCCS San Raffaele Pisana Rome Italy; ^9^ University of Warwick Coventry UK; ^10^ Department of Cardiology (CVK) and Berlin Institute of Health Center for Regenerative Therapies (BCRT), German Centre for Cardiovascular Research (DZHK), partner site Berlin Charité Universitätsmedizin Berlin Augustenburger Platz 1 D‐13353 Berlin Germany

## Abstract

**Aims:**

We sought to conduct a meta‐analysis regarding the safety and efficacy of sodium‐glucose co‐transporter 2 (SGLT2) inhibitors in patients with heart failure (HF).

**Methods and results:**

MEDLINE, Scopus, Cochrane CENTRAL, and ClinicalTrials.gov were searched from their inception to November 2020 for placebo‐controlled randomized controlled trials of SGLT2 inhibitors. Randomized controlled trials were selected if they reported at least one of the prespecified outcomes in patients with HF. Hazard ratios (HRs) or risk ratios and their corresponding 95% confidence intervals were pooled using a random‐effects model. A total of seven trials including 16 820 HF patients (*N* = 8884 in the SGLT2 inhibitor arms; *N* = 7936 in the placebo arms) were included. In the overall HF cohort, SGLT2 inhibitors compared with placebo significantly reduced the risk of the composite endpoint of first HF hospitalization or cardiovascular death [HR: 0.77 (0.72–0.83); *P* < 0.001; *I* ^2^ = 0%], time to first HF hospitalization [HR: 0.71 (0.64–0.78); *P* < 0.001; *I* ^2^ = 0], cardiovascular mortality [HR: 0.87 (0.79–0.96); *P* = 0.005; *I* ^2^ = 0%], and all‐cause mortality [HR: 0.89 (0.82–0.96); *P* = 0.004; *I* ^2^ = 0%]. Results remained consistent across HF‐specific trials and according to diabetes mellitus status. A trend towards benefit was observed in patients with HF with preserved ejection fraction for the composite of HF hospitalization and cardiovascular death [HR: 0.80 (0.63–1.00); *P* = 0.05; *I* ^2^ = 29%]. No increased risk of hypovolaemia, hyperkalaemia, and hypotension was seen with SGLT2 inhibitors compared with placebo.

**Conclusions:**

SGLT2 inhibitors significantly improve cardiovascular outcomes including cardiovascular and all‐cause mortality in patients with HF without an increased risk of serious adverse events. A trend towards benefit was observed in patients with HF with preserved ejection fraction.

## Introduction

Sodium‐glucose co‐transporter 2 (SGLT2) inhibitors have emerged as a new foundational therapy in patients with heart failure (HF) and reduced ejection fraction (HFrEF).^1^ Large‐scale randomized controlled trials (RCTs) of SGLT2 inhibitors have shown significant cardiovascular and renal benefit across various subgroups.^2^, ^3^, ^4^, ^5^ Three RCTs have specifically assessed the effects of SGLT2 inhibitors in the HF population. The DAPA‐HF (Dapagliflozin and Prevention of Adverse‐Outcomes in Heart Failure) trial and EMPEROR‐Reduced (EMPagliflozin outcomE tRial in Patients With chrOnic heaRt Failure With Reduced Ejection Fraction) trial evaluated the effects of dapagliflozin and empagliflozin, respectively, in patients with HFrEF.^6^, ^7^ The recently published SOLOIST‐WHF (Effect of Sotagliflozin on Cardiovascular Events in Patients With Type 2 Diabetes Post Worsening Heart Failure) trial studied the effects of sotagliflozin (a combined SGLT2/SGLT1 inhibitor) in a population comprising both patients with HFrEF and patients with HF and preserved ejection fraction (HFpEF).^8^ All three trials found a significant reduction in the composite endpoint of first HF hospitalization or cardiovascular death. These results are congruent with data from HF subgroups of cardiovascular outcomes amongst patients with type 2 diabetes mellitus (DM).

SGLT2 inhibitor trials were not powered to study cardiovascular and all‐cause mortality. Moreover, previous meta‐analyses of SGLT2 inhibitors have not specifically focused on patients with prevalent HF across all trials, and none of the studies have included results from the recently published SOLOIST‐WHF trial.^9^, ^10^, ^11^, ^12^, ^13^ Herein, we present an updated comprehensive meta‐analysis of SGLT2 inhibitors in HF patients, stratified by HFrEF and HFpEF.

## Methods

### Data sources and search strategy

This meta‐analysis was conducted and reported in conformity with the Cochrane and PRISMA (Preferred Reporting Items for Systematic review and Meta‐Analyses) guidelines.^14^, ^15^ An electronic search of the MEDLINE, Scopus, and Cochrane CENTRAL databases was conducted from their inception up until 18 November 2020. No language restrictions were set. The following keywords and their MeSH terms were used for the search: ‘(sodium‐glucose co‐transporter inhibitor OR SGLT2 inhibitor OR SGLT‐2 inhibitor OR SGLT 2 inhibitor OR tofogliflozin OR sotagliflozin OR empagliflozin OR canagliflozin OR dapagliflozin OR ertugliflozin OR luseugliflozin OR ipragliflozin OR remogliflozin OR sergliflozin) AND (heart failure or cardiac failure OR CHF)’. The detailed search strategy for each database is presented in the Supporting Information, *Table* *S1*. We also searched ClinicalTrials.gov for completed RCTs using pharmaceutical, generic, and trade names of all SGLT2 inhibitors. Reference lists of included trials were manually screened to identify any relevant studies that may have been missed during the search.

### Study selection and eligibility criteria

Articles retrieved from the systematic search were exported to EndNote Reference Library software, and duplicates were removed. The remaining articles were first screened on the basis of title and abstract, after which the full text was reviewed to assess relevance. Screening of articles was conducted independently by two reviewers (M. S. U. and M. S. K.). Any discrepancies were resolved by discussion till consensus. Articles were selected for inclusion if they met all the following prespecified eligibility criteria: (i) compared SGLT2 inhibitors with placebo; (ii) included HF patients; (ii) were either RCTs or post hoc analyses of RCTs; and (iv) reported at least one of the predefined outcomes of interest.

### Outcomes of interest, data extraction, and quality assessment

The main outcome of interest was the composite of HF hospitalization or cardiovascular death. Other outcomes of interest were (i) composite of total (first and recurrent) HF hospitalization and cardiovascular death; (ii) first HF hospitalization; (iii) cardiovascular death; (iv) all‐cause death; and (v) a composite renal endpoint, which was defined as a >40% sustained decline in estimated glomerular filtration rate, end‐stage renal disease, or renal death. The proportion of total serious adverse events and serious adverse events leading to study drug discontinuation were also evaluated. Incidence of the following specific adverse events was also studied: volume depletion, hyperkalaemia, hypotension, major hypoglycaemia, amputations, bone fractures, acute kidney injury, and urinary tract infections. Study characteristics, baseline demographics, outcome data, and safety data were extracted onto a predesigned Excel spreadsheet. Quality assessment of included RCTs was conducted using the Cochrane Risk of Bias tool.^16^ Data extraction and quality assessment were conducted independently by two reviewers (M. S. U. and M. S. K.), and discrepancies were resolved via discussion.

### Statistical analysis

Review Manager (Version 5.3, Copenhagen: The Nordic Cochrane Centre, The Cochrane Collaboration, 2014) was used for all statistical analyses. For time‐to‐event clinical outcomes, hazard ratios (HRs) and corresponding 95% confidence intervals from each trial were extracted. These were transformed to Ln(HR) and standard errors and pooled using a random‐effects model. Standard errors were calculated using the formula outlined in the Cochrane handbook section 7.7.7.2.^15^ Instead of HRs, rate ratios were used for recurrent outcomes. For adverse events, raw data were used to calculate risk ratios (RRs) and 95% confidence intervals, which were then meta‐analysed using a random‐effects model. A random‐effects model was selected to account for expected heterogeneity in study design, study drug and dosage, and certain outcome definitions.^17^ Study weights were assigned using an inverse variance method. As post hoc subgroup analyses of HF patients from cardiovascular and kidney outcome trials are likely to have lower power, less rigorous HF adjudication, and more risk of bias than trials designed specifically to study HF patients; HF‐specific trials were also analysed separately.

The overall HF population included patients with HFrEF and HFpEF and patients with unknown type of HF. If results were reported by HF subtype, the HFrEF and HFpEF populations were analysed separately as well. For the overall HF cohort and HFrEF cohort, subgroup analysis based on DM status was performed for all outcomes. For the DAPA‐HF and EMPEROR‐Reduced trials, data of diabetes subgroups were taken from post hoc studies.^18^, ^19^ Subgroup analyses based on age, sex, and angiotensin receptor/neprilysin inhibitor use were conducted only for the HFrEF population, as data for these subgroups were only available for this population. The *χ* ^2^ test was used to test for subgroup differences. Statistical heterogeneity was evaluated using the Higgins *I* ^2^ statistic.^20^ Publication bias could not be evaluated visually in funnel plots as less than 10 studies were identified. A *P*‐value ≤0.05 was considered statistically significant.

## Results

### Search results, study characteristics, and baseline demographics

The PRISMA flow chart (Supporting Information, *Figure S1*) summarizes the search and study selection process. Data from seven RCTs evaluating SGLT2 inhibitors were included. This included three HF‐specific trials (DAPA‐HF, EMPEROR‐Reduced, and SOLOIST‐WHF)^6^, ^7^, ^8^ and four cardiovascular outcome trials: CANVAS (CANagliflozin cardioVascular Assessment Study),^3^ EMPA‐REG OUTCOME (Empagliflozin Cardiovascular Outcome Event Trial in Type 2 Diabetes Mellitus Patients),^4^ DECLARE‐TIMI 58 (Multicenter Trial to Evaluate the Effect of Dapagliflozin on the Incidence of Cardiovascular Events),^5^ and VERTIS‐CV (eValuation of ERTugliflozin effIcacy and Safety CardioVascular outcomes trial).^21^ HF patient data from the cardiovascular outcome trials were available from published post hoc studies.^22^, ^23^, ^24^, ^25^ Although the recently published SCORED (Cardiovascular and Renal Events in Patients With Type 2 Diabetes and Moderate Renal Impairment Who Are at Cardiovascular Risk) trial did not report outcome data of patients with baseline HF separately,^26^ the authors of this trial presented a pooled analysis of the SOLOIST‐WHF and SCORED trials at the American Heart Association 2020 conference. Whenever possible, the pooled findings from these trials were used in our analysis.

The overall study population in this meta‐analysis included 16 820 HF patients (*n* = 8884 in the SGLT2 inhibitor arms; *n* = 7936 in the placebo arms). The HFrEF subpopulation composed of 11 381 patients (*n* = 5750 in the SGLT2 arms; *n* = 5631 in the placebo arms). The HFpEF subpopulation composed of 2554 patients (*n* = 1447 in the SGLT2 arm; *n* = 1107 in the placebo arm). The rest of the patients had unknown type of HF. *Table* *1* shows the baseline characteristics of the included studies. The CANVAS, EMPA‐REG OUTCOME, DECLARE‐TIMI 58, VERTIS‐CV, and SOLOIST‐WHF trials included only DM patients. The DAPA‐HF and EMPEROR‐Reduced trials included both DM and non‐DM patients. DAPA‐HF and EMPEROR‐Reduced included only HFrEF patients, while all other trials included both HFrEF patients and HFpEF patients. Three trials (SOLOIST‐WHF, VERTIS‐CV, and DECLARE‐TIMI 58) reported data of patients with HFpEF separately. In VERTIS‐CV and DECLARE‐TIMI 58, an ejection fraction >45% with known HF was considered as HFpEF, while in SOLOIST‐WHF, an ejection fraction >50% was considered HFpEF. All included trials were at low risk of bias (Supporting Information, *Table* *S2*). Although the SOLOIST‐WHF trial was stopped prematurely due to loss of funding, this is unlikely to be a major source of bias. In this trial, investigator‐reported events (not adjudicated events) were reported, which may have some risk of bias.

**Table 1:** Study and baseline characteristics

	EMPEROR‐Reduced		DAPA‐HF		CANVAS		SOLOIST‐WHF		VERTIS‐CV		EMPA‐REG OUTCOME		DECLARE‐TIMI 58^a^	
	Empa	Plac	Dapa	Plac	Cana	Plac	Sota	Plac	Ertu	Plac	Empa	Plac	Dapa	Plac
Number of participants	1863	1867	2373	2371	803	658	608	614	1286	672	462	244	852	872
Age, years (SD)	67.2 (10.8)	66.5 (11.2)	66.2 (11.0)	66.5 (10.8)	64.1 (8.3)	63.4 (8.3)	69 (63–76)	70 (64–76)	64.2 (7.9)	64.7 (7.8)	64.5 (8.8)	64.5 (8.9)		
Sex, *n* (%)														
Men	1426 (76.5)	1411 (75.6)	1809 (76.2)	1826 (77.0)	457 (56.9)	356 (54.1)	410 (67.4)	400 (65.1)	891 (69.3)	443 (65.9)	320 (69.3)	175 (71.7)		
Women	437 (23.5)	456 (24.4)	564 (23.8)	545 (23.0)	346 (43.1)	302 (45.9)	198 (32.6)	214 (34.9)	395 (30.7)	229 (34.1)	142 (30.7)	69 (28.3)		
NYHA functional classification, %														
II	75.1	75.0	67.7	67.4					62.0	67.1				
III	24.4	24.4	31.5	31.7					5.5	3.8				
IV	0.5	0.6	0.8	1.0					0.0	0.0				
Mean LVEF, %	27.7 (6.0)	27.2 (6.1)	31.2 (6.7)	30.9 (6.9)			35 (28–47)	35 (28–45)						
HFpEF, %							127 (20.9)	129 (21.0)	680 (52.9)	327 (48.6)			399 (46.8)	409 (46.9)
HFrEF, %							481 (79.1)	485 (79.0)	319 (24.8)	159 (23.7)			318 (37.3)	353 (40.4)
NT‐proBNP, pg/mL	1887 (1077–3429)	1926 (1153–3525)	1428 (857–2655)	1446 (857–2641)			1817 (845–3659)	1741 (843–3582)						
Hospitalization for heart failure	577 (31.0)	574 (30.7)	1124 (47.4)	1127 (47.5)										
Diabetes^b^	927 (49.8)	929 (49.8)	1075 (45.3)	1064 (44.9)	803 (100%)	658 (100%)	25 (4.1)	20 (3.3)	1286 (100)	672 (100)	462 (100)	244 (100)		
Duration of diabetes, years					11.9 (7.9)	12.2 (7.7)			11.9 (8.0)	12.3 (7.8)				
eGFR, mL/min/1.73 m^2^ ^d^	61.8 (21.7)	62.2 (21.5)	66.0 (19.6)	65.5 (19.3)	72.7 (19.5)	73.3 (19.8)	49.2 (39.5–61.2)	50.5 (40.5–64.6)			68.4 (20.2)	69.3 (20.7)		
Heart failure medications, *n* (%)														
ACE inhibitor	867 (46.5)	836 (44.8)	1332 (56.1)	1329 (56.1)	680 (84.7)	572 (86.9)	254 (41.8)	241 (39.3)	1078 (83.8)	573 (85.3)	406 (87.9)	206 (84.4)		
ARB	451 (24.2)	457 (24.5)	675 (28.4)	632 (26.7)			245 (40.3)	270 (44.0)						
Mineralocorticoid receptor antagonist	1306 (70.1)	1355 (72.6)	1696 (71.5)	1674 (70.6)			403 (66.3)	385 (62.7)	253 (19.7)	113 (16.8)	116 (25.1)	53 (21.7)		
ARNI	340 (18.3)	387 (20.7)	250 (10.5)	258 (10.9)			93 (15.3)	112 (18.2)						
ICD or CRT‐D	578 (31.0%)	593 (31.8%)	622 (26.2%)	620 (26.1%)										
CRT‐D or CRT‐P	220 (11.8%)	222 (11.9%)	190 (8.0%)	164 (6.9%)										

ACE, angiotensin‐converting enzyme; ARB, angiotensin receptor blocker; ARNI, angiotensin receptor/neprilysin inhibitor; CRT‐D, cardiac resynchronization therapy defibrillator; CRT‐P, cardiac resynchronization therapy pacemaker; eGFR, estimated glomerular filtration rate; HFpEF, heart failure with preserved ejection fraction; HFrEF, heart failure with reduced ejection fraction; ICD, implantable cardiac defibrillator; LVEF, left ventricular ejection fraction; NT‐proBNP, N‐terminal pro‐B‐type natriuretic peptide; NYHA, New York Heart Association; SD, standard deviation.

Data are reported as *n* (%), mean (SD), or median (interquartile range).

^a^ Aggregate baseline data reported of patients with HF on dapagliflozin and placebo regimen.

^b^ For SOLOIST‐WHF, within 3 months before randomization. For EMPEROR‐REDUCED and DAPA‐HF, a combination of medical history and pretreated glycated haemoglobin.

^c^ For VERTIS‐CV and DECLARE‐TIMI 58, an ejection fraction <45% with known HF was considered as HFrEF; for SOLOIST‐WHF, an ejection fraction <50% was considered HFrEF.

^d^ Determined by Chronic Kidney Disease Epidemiology Collaboration formula.

### Results for the overall heart failure cohort

#### First heart failure hospitalization or cardiovascular death (*Figure* *1* *A*)

**Figure 1:**
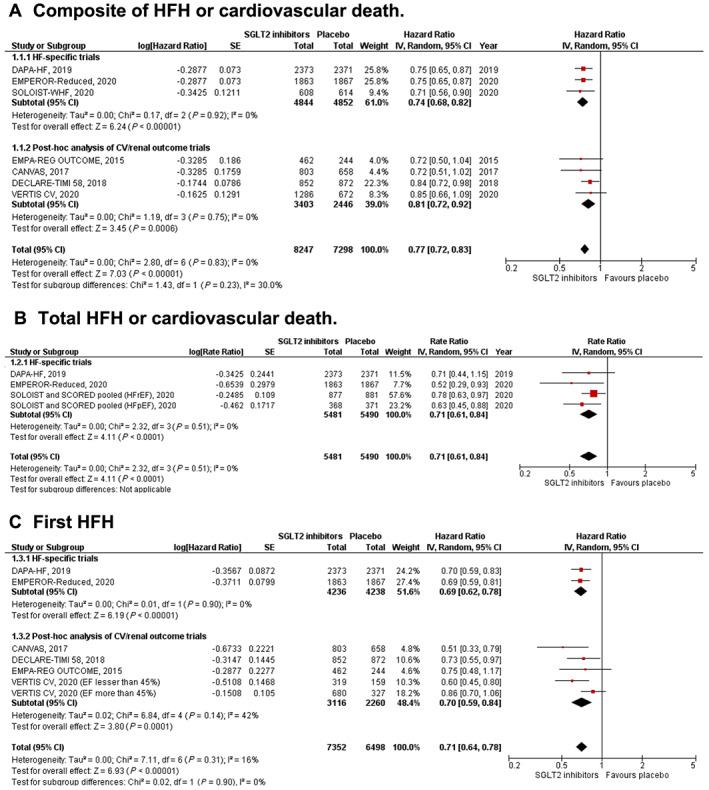
Forest plot displaying the effects of sodium‐glucose co‐transporter 2 (SGLT2) inhibitors vs. placebo in an overall cohort of heart failure (HF) patients, for the following outcomes: (A) composite of first HF hospitalization (HFH) or cardiovascular death; (B) total (first and recurrent) HFH or cardiovascular death; (C) first HFH; (D) cardiovascular death; (E) all‐cause death; and (F) renal composite.

SGLT2 inhibitors significantly reduced the composite of first HF hospitalization or cardiovascular death [HR: 0.77 (0.72–0.83); *P* < 0.001; *I* ^2^ = 0%]. The findings from HF‐specific trials and post hoc analyses were consistent (*P*‐value for subgroup differences = 0.23).

#### Total heart failure hospitalization or cardiovascular death (*Figure* *1* *B*)

SGLT2 inhibitors resulted in a significant reduction in total HF hospitalizations and cardiovascular death [rate ratio: 0.71 (0.61–0.84); *P* < 0.001; *I* ^2^ = 0%]. This outcome was only reported by HF‐specific trials.

#### First heart failure hospitalization (*Figure* *1* *C*)

SGLT2 inhibitors were associated with a significant reduction in first HF hospitalization [HR: 0.71 (0.64–0.78); *P* < 0.001; *I* ^2^ = 0%]. This finding was seen in both HF‐specific trials and post hoc subgroup studies (*P*‐value for subgroup differences = 0.90).

#### Cardiovascular death (*Figure* *1* *D*)

SGLT2 inhibitors significantly reduced the incidence of cardiovascular death [HR: 0.87 (0.79–0.96); *P* = 0.005; *I* ^2^ = 0%]. The findings were consistent in HF‐specific trials and post hoc studies (*P*‐value for subgroup differences = 0.97).

#### All‐cause death (*Figure* *1* *E*)

SGLT2 inhibitors significantly reduced the risk of all‐cause death in the overall HF cohort compared with placebo [HR: 0.89 (0.82–0.96); *P* = 0.004; *I* ^2^ = 0%]. HF‐specific trials and post hoc studies of cardiovascular trials showed similar results on subgroup analyses (*P*‐value for subgroup differences = 0.44).

#### Renal composite (*Figure* *1* *F*)

The occurrence of the composite renal endpoint was lower with SGLT2 inhibitor use [HR: 0.60 (0.48–0.75); *P* < 0.001; *I* ^2^ = 0%]. This finding was consistent in both HF‐specific trials and post hoc analyses of cardiovascular trials (*P*‐value for subgroup differences = 0.80).

### Heart failure with reduced ejection fraction

#### First heart failure hospitalization or cardiovascular death (*Figure* *2* *A*)

**Figure 2:**
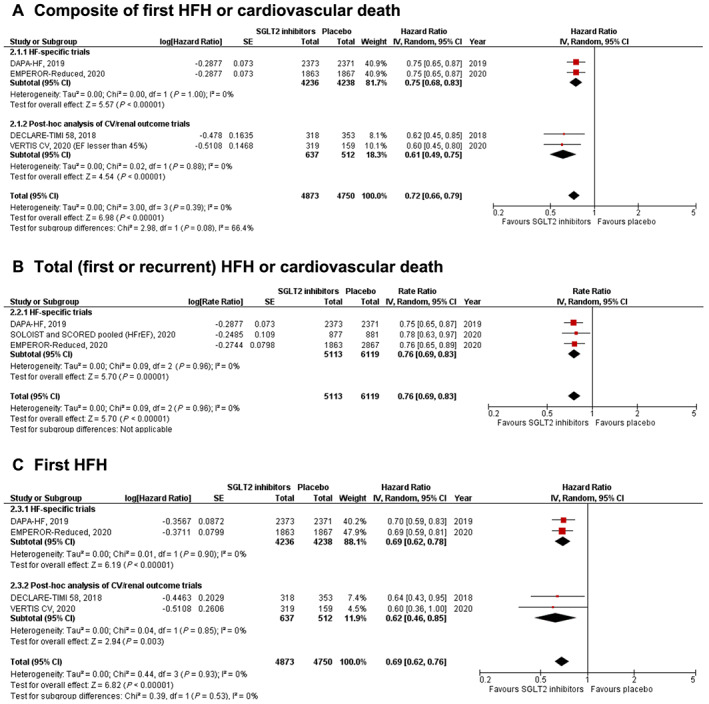
Forest plot displaying the effects of sodium‐glucose co‐transporter 2 (SGLT2) inhibitors vs. placebo in patients with heart failure (HF) with reduced ejection fraction, for the following outcomes: (A) composite of first HF hospitalization (HFH) or cardiovascular death; (B) total (first and recurrent) HFH or cardiovascular death; (C) first HFH; (D) cardiovascular death; (E) all‐cause death; and (F) renal composite.

SGLT2 inhibitors resulted in a significant reduction in the composite of first HF hospitalization or cardiovascular death [HR: 0.72 (0.66–0.79); *P* < 0.001; *I* ^2^ = 0%]. The findings from HF‐specific trials and post hoc studies were consistent (*P*‐value for subgroup differences = 0.08).

#### Total heart failure hospitalizations or cardiovascular death (*Figure* *2* *B*)

SGLT2 inhibitors resulted in a significant reduction in total HF hospitalization or cardiovascular death [rate ratio: 0.76 (0.69–0.83); *P* < 0.001; *I* ^2^ = 0%]. This outcome was only reported by HF‐specific trials.

#### First heart failure hospitalization (*Figure* *2* *C*)

SGLT2 inhibitors were associated with a significant reduction in first HF hospitalization [HR: 0.69 (0.62–0.76); *P* < 0.001; *I* ^2^ = 0%]. This finding was seen in both HF‐specific trials and post hoc subgroup studies (*P*‐value for subgroup differences = 0.53).

#### Cardiovascular death (*Figure* *2* *D*)

SGLT2 inhibitors significantly reduced the incidence of cardiovascular death in HFrEF patients [HR: 0.83 (0.71–0.98); *P* = 0.03; *I* ^2^ = 26%]. The findings were consistent in HF‐specific trials and post hoc studies (*P*‐value for subgroup differences = 0.53).

#### All‐cause death (*Figure* *2* *E*)

SGLT2 inhibitors significantly reduced the risk of all‐cause death in the overall HF cohort, when compared with placebo [HR: 0.84 (0.72–0.97); *P* = 0.02; *I* ^2^ = 32%]. HF‐specific trials and post hoc studies of cardiovascular trials were similar for this result (*P*‐value for subgroup differences = 0.48).

#### Renal composite (*Figure* *2* *F*)

The occurrence of the composite renal endpoint was lower with SGLT2 inhibitor use compared with placebo [HR: 0.63 (0.43–0.91); *P* < 0.001; *I* ^2^ = 0%]. This finding was only reported by HF‐specific trials.

### Heart failure with preserved ejection fraction

#### Heart failure hospitalization or cardiovascular death (*Figure* *3* *A*)

**Figure 3:**
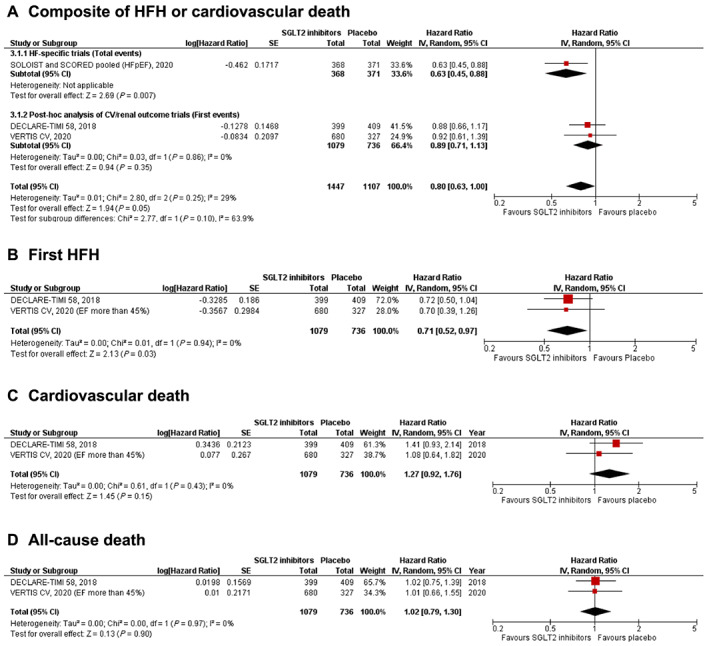
Forest plot displaying the effects of sodium‐glucose co‐transporter 2 (SGLT2) inhibitors vs. placebo in patients with heart failure (HF) with preserved ejection fraction, for the following outcomes: (A) composite of HF hospitalization (HFH) or cardiovascular death; (B) first HFH; (C) cardiovascular death; and (D) all‐cause death.

Data from the DECLARE‐TIMI 58 trial and VERTIS CV trial were combined with pooled data from SOLOIST‐WHF and SCORED trials for this outcome. A borderline significant reduction in the composite of HF hospitalization or cardiovascular death was seen with SGLT2 inhibitor use [HR: 0.80 (0.63–1.00); *P* = 0.05; *I* ^2^ = 29%]. However, this analysis was only exploratory, as the outcome reported for the SOLOIST‐WHF and SCORED trials (*total* HF hospitalization or cardiovascular death) was different from the outcome reported by the other two trials (*first* HF hospitalization or cardiovascular death). Upon sensitivity analysis by excluding SOLOIST‐WHF/SCORED data, the results became non‐significant but continued to trend towards a benefit with SGLT2 inhibitors [HR: 0.89 (0.71–1.13); *P* = 0.35; *I* ^2^ = 0%].

#### First heart failure hospitalization (*Figure* *3* *B*)

This outcome was reported by two post hoc studies from DECLARE‐TIMI 58 and VERTIS‐CV. A significant reduction in first HF hospitalization was seen with SGLT2 inhibitors [HR: 0.71 (0.52–0.97); *P* = 0.03; *I* ^2^ = 0%].

#### Cardiovascular death (*Figure* *3* *C*)

This outcome was reported by two post hoc studies from DECLARE‐TIMI 58 and VERTIS‐CV. There was no significant difference between the SGLT2 inhibitor and placebo groups in the incidence of cardiovascular death amongst HFpEF patients [HR: 1.27 (0.92–1.76); *P* = 0.15; *I* ^2^ = 0%].

#### All‐cause death *Figure* *3* *D*)

This outcome was reported by two post hoc studies from DECLARE‐TIMI 58 and VERTIS‐CV. There was no significant difference between the SGLT2 inhibitor arm and the placebo arm in the incidence of all‐cause death [HR: 1.27 (0.92–1.76); *P* = 0.15; *I* ^2^ = 0%].

### Subgroup analysis

The detailed forest plots displaying subgroup analyses are presented in the Supporting Information. For all outcomes in the overall HF population (Supporting Information, *Figure* *S2*) as well as the HFrEF population (Supporting Information, *Figure* *S3*), no subgroup effect was seen upon stratification by DM status. Similarly, age (*P*‐value for subgroup differences = 0.55), sex (*P*‐value for subgroup differences = 0.49), and angiotensin receptor/neprilysin inhibitor use (*P*‐value for subgroup differences = 0.52) were not found to modify the treatment effect on the composite endpoint of first HF hospitalization or cardiovascular death amongst the HFrEF population.

### Safety

In the overall cohort, the incidence of treatment‐emergent serious adverse events [RR: 0.88 (0.84–0.91); *P* < 0.001; *I* ^2^ = 0%] and risk of acute kidney injury [RR: 0.63 (0.45–0.87); *P* = 0.006; *I* ^2^ = 14%] was significantly lower in the SGLT2 inhibitor arm. There was no significant difference between the SGLT2 inhibitor and placebo groups in the rate of discontinuation due to adverse events [RR: 1.07 (0.91–1.25); *P* = 0.40; *I* ^2^ = 0%]. SGLT2 inhibitors did not significantly increase the risk of volume depletion [RR: 1.11 (0.98–1.25); *P* = 0.11; *I* ^2^ = 0%], hypotension [RR: 1.05 (0.84–1.32); *P* = 0.65; *I* ^2^ = 0%], hyperkalaemia [RR: 0.79 (0.49–1.29); *P* = 0.35; *I* ^2^ = 0%], major hypoglycaemia [RR: 1.09 (0.84–1.42); *P* = 0.53; *I* ^2^ = 9%], bone fractures [RR: 1.11 (0.89–1.39); *P* = 0.36; *I* ^2^ = 0%], and urinary tract infections [RR: 1.08 (0.87–1.35); *P* = 0.48; *I* ^2^ = 0%]. SGLT2 inhibitor use was associated with a significant increase in the risk of amputations [RR: 1.57 (1.04–2.35); *P* = 0.03; *I* ^2^ = 0%].

## Discussion

In this comprehensive analysis including almost 17 000 HF patients, SGLT2 inhibitors significantly reduced the risk of all‐cause mortality, cardiovascular mortality, HF hospitalization, and renal outcomes. These findings remained consistent when the HFrEF population was analysed separately and also when the patients were stratified according to DM status. A trend towards better HF hospitalization‐related outcomes was seen in the HFpEF subpopulation; however, this finding should be considered exploratory rather than definitive due to availability of scarce and heterogeneous data. In comparison with placebo, SGLT2 inhibitors were not found to increase the risk of serious adverse events or discontinuation due to adverse events.

Important differences between the three HF‐specific trials must be kept in mind. The EMPEROR‐Reduced trial enrolled sicker patients with higher N‐terminal pro‐B‐type natriuretic peptide concentrations and lower estimated glomerular filtration rate. The SOLOIST‐WHF trial randomized patients with DM and HF irrespective of ejection fraction who were admitted for worsening HF to either sotagliflozin or placebo.^8^ Sotagliflozin differs from other SGLT2 inhibitors given its additional SGLT1 inhibiting properties. The remarkable consistency across the three trials for all outcomes evaluated in this meta‐analysis is reassuring, yet more data are necessary for patients with HFpEF. Furthermore, post hoc analyses of cardiovascular trials are also consistent with HF‐specific trials, further reinforcing the efficacy of SGLT2 inhibitors.

All three HF‐specific trials showed significant reductions in the composite of first HF hospitalization or cardiovascular death. While DAPA‐HF trial showed a nominally significant reduction in cardiovascular and all‐cause mortality, EMPEROR‐Reduced and SOLOIST‐WHF did not. However, none of these trials were powered to study mortality. Meta‐analysis of these trials demonstrates a significant reduction and cardiovascular and all‐cause mortality. Results from post hoc analysis of cardiovascular outcome trials further support the mortality benefit in HF patients. Our findings also indicate that the mortality benefit persists regardless of DM status.

Unlike HFrEF, the effects of SGLT2 inhibition in HFpEF patients remain unclear. Current evidence with SGLT2 inhibitors, albeit limited, is encouraging. Combined results from the SOLOIST‐WHF and SCORED trials, as well as findings from subgroup analyses of VERTIS‐CV and DECLARE‐TIMI 58, suggest potential reductions in the composite of HF hospitalization or cardiovascular death. Two ongoing SGLT2 inhibitor trials [EMPEROR‐PRESEVED (EMPagliflozin outcomE tRial in Patients With chrOnic heaRt Failure With Preserved Ejection Fraction) (NCT03057951) and DELIVER (Dapagliflozin Evaluation to Improve the LIVEs of Patients with PReserved Ejection Fraction Heart Failure) (NCT01297257)] will provide further clarity.

There are some limitations in this analysis. The analysis for the HFpEF cohort should only be viewed as exploratory, as this analysis was underpowered, and the data were heterogeneous. In particular, for the composite outcome of HF hospitalization or cardiovascular death, recurrent event data from the SOLOIST‐WHF and SCORED trials were pooled with time‐to‐first‐event data from the VERTIS‐CV and DECLARE‐TIMI 58 trials. While these data are distinct, simulations have shown that point estimates for both are usually similar.^27^ The definition of HFpEF varied across trials, with VERTIS‐CV and DECLARE‐TIMI 58 using a left ventricular ejection fraction >45% as a cut‐off for HFpEF, while the SOLOIST‐WHF/SCORED trials used a threshold of left ventricular ejection fraction >50%. Due to these differences, a sensitivity analysis by removing SOLOIST‐WHF/SCORED data was also conducted. We did not do a correction for multiplicity of subgroup testing; hence, our subgroup analysis should be considered hypothesis generating. Finally, although a random‐effects model was used to account for methodological heterogeneity, certain differences across studies may limit interpretation. This includes the use of different SGLT2 inhibitor subtypes, varied proportions of men and women, differences in background therapy, baseline risk severity of patients enrolled, and discrepancies in the definition of safety and renal endpoints across trials.

In conclusion, this updated meta‐analysis of ~17 000 HF patients shows that SGLT2 inhibitors significantly decrease HF hospitalizations, adverse renal outcomes, and mortality in patients with HF. These findings remained consistent in patients with HFrEF and in both diabetics and non‐diabetics. Encouraging trend for benefit was observed in patients with HFpEF. Our findings reinforce that all patients with HF, regardless of DM status, may benefit from SGLT2 inhibitors.

## Conflict of interest

J.B. is a consultant for Abbott, Amgen, Applied Therapeutics, AstraZeneca, Bayer, Boehringer Ingelheim, Bristol Myers Squib, CVRx, Janssen, LivaNova, Luitpold, Medtronic, Merck, Novartis, Relypsa, and Vifor. S.J.G. was supported by Heart Failure Society of America/Emergency Medicine Foundation Acute Heart Failure Young Investigator Award funded by Novartis, has received research support from Amgen, Bristol‐Myers Squibb, and Novartis, and serves on an advisory board for Amgen. M.V. is supported by the KL2/Catalyst Medical Research Investigator Training award from Harvard Catalyst (NIH/NCATS Award UL 1TR002541), serves on advisory boards for Amgen, AstraZeneca, Baxter Healthcare, Bayer AG, Boehringer Ingelheim, Cytokinetics, and Relypsa, and participates on clinical endpoint committees for studies sponsored by Galmed, Novartis, and the NIH. T.F. reports personal fees from Novartis, Bayer, Janssen, SGS, Roche, Boehringer Ingelheim, Daiichi‐Sankyo, Galapagos, Penumbra, Parexel, Vifor, BiosenseWebster, CSL Behring, Fresenius Kabi, Coherex Medical, and LivaNova. G.F. participated in committees of trials and registries sponsored by BI, Bayer, Medtronic, Servier, Novartis, and Vifor. A.J.S.C. reports honoraria and/or lecture fees from AstraZeneca, Bayer, Menarini, Novartis, Nutricia, Servier, Vifor, Actimed, Cardiac Dimensions, CVRx, Enopace, Faraday, Gore, Impulse Dynamics, Respicardia, Stealth Peptides, V‐Wave, Corvia, Arena, and ESN Cleer. S.D.A. has received research support from Vifor International & Abbott Vascular and fees for consultancy and/or speaking from Astra‐Zeneca, Bayer, Boehringer Ingelheim, Respicardia, Impulse Dynamics, Janssen, Novartis, Servier, and Vifor International. All other authors report no disclosures.

## Supporting information


**Table S1.** Detailed search strategy for each database.
**Table S2.** Quality Assessment of included trials.
**Figure S1.** PRISMA flow chart.
**Figure S2.** Forest plots displaying subgroup analysis according to DM status in the overall cohort of HF patients for the following outcomes: (A) composite of first HFH or cardiovascular death*; (B) total (first and recurrent) HFH or cardiovascular death; (C) First HFH; (D) cardiovascular death; (E) all‐cause death; (F) Renal composite.
**Figure S3.** Forest plots displaying subgroup analysis according to DM status in the cohort of HFrEF patients for the following outcomes: (A) composite of first HFH or cardiovascular death*; (B) total (first and recurrent) HFH or cardiovascular death; (C) First HFH; (D) cardiovascular death; (E) all‐cause death; (F) Renal composite.
