# Corrigendum

**DOI:** 10.1002/ehf2.13338

**Published:** 2021-05-01

In the paper by Butler *et al*.^1^ panels D to F in both Figures 1 and 2 were erroneously omitted. The last sentence in the results section under safety heading “SGLT2 was associated with increased risk of amputations” was also wrongly included. Both these errors have been corrected in the online published version of the issue.
