# Supplementary material for: Efficacy and safety of SGLT2 inhibitors in heart failure: systematic review and meta‐analysis
Source: ESC Heart Fail. 2020 Dec 22;7(6):3298–309. doi: 10.1002/ehf2.13169 (PMC7755023; doi:10.1002/ehf2.13169)
Supplement: Supplementary file 1 — Table S1. Detailed search strategy for each database. Table S2. Quality Assessment of included trials. Figure S1. PRISMA flow chart. Figure S2. Forest plots displaying subgroup analysis according to DM status in the overall cohort of HF patients for the following outcomes: (A) composite of first HFH or cardiovascular death*; (B) total (first and recurrent) HFH or cardiovascular death; (C) First HFH; (D) cardiovascular death; (E) all‐cause death; (F) Renal composite. Figure S3. Forest plots displaying subgroup analysis according to DM status in the cohort of HFrEF patients for the following outcomes: (A) composite of first HFH or cardiovascular death*; (B) total (first and recurrent) HFH or cardiovascular death; (C) First HFH; (D) cardiovascular death; (E) all‐cause death; (F) Renal composite. [file EHF2-7-3298-s001.DOCX]

**SUPPLEMENTARY MATERIAL**

**Supplemental table 1: Detailed search strategy for each database**

| **Database** | **String** | **Results** |
| --- | --- | --- |
| Medline | (("sodium-glucose"[All Fields] AND "co-transporter"[All Fields] AND ("antagonists and inhibitors"[MeSH Subheading] OR ("antagonists"[All Fields] AND "inhibitors"[All Fields]) OR "antagonists and inhibitors"[All Fields] OR "inhibitors"[All Fields] OR "inhibitor"[All Fields] OR "inhibitor s"[All Fields])) OR ("sodium glucose transporter 2 inhibitors"[Pharmacological Action] OR "sodium glucose transporter 2 inhibitors"[MeSH Terms] OR "sodium glucose transporter 2 inhibitors"[All Fields] OR ("sglt2"[All Fields] AND "inhibitor"[All Fields]) OR "sglt2 inhibitor"[All Fields]) OR ("SGLT-2"[All Fields] AND ("antagonists and inhibitors"[MeSH Subheading] OR ("antagonists"[All Fields] AND "inhibitors"[All Fields]) OR "antagonists and inhibitors"[All Fields] OR "inhibitors"[All Fields] OR "inhibitor"[All Fields] OR "inhibitor s"[All Fields])) OR ("SGLT"[All Fields] AND "2"[All Fields] AND ("antagonists and inhibitors"[MeSH Subheading] OR ("antagonists"[All Fields] AND "inhibitors"[All Fields]) OR "antagonists and inhibitors"[All Fields] OR "inhibitors"[All Fields] OR "inhibitor"[All Fields] OR "inhibitor s"[All Fields])) OR ("6 4 ethylphenyl methyl 3 4 5 6 tetrahydro 6 hydroxymethyl spiro isobenzofuran 1 3h 2 2h pyran 3 4 5 triol"[Supplementary Concept] OR "6 4 ethylphenyl methyl 3 4 5 6 tetrahydro 6 hydroxymethyl spiro isobenzofuran 1 3h 2 2h pyran 3 4 5 triol"[All Fields] OR "tofogliflozin"[All Fields]) OR ("2s 3r 4r 5s 6r 2 4 chloro 3 4 ethoxybenzyl phenyl 6 methylthio tetrahydro 2h pyran 3 4 5 triol"[Supplementary Concept] OR "2s 3r 4r 5s 6r 2 4 chloro 3 4 ethoxybenzyl phenyl 6 methylthio tetrahydro 2h pyran 3 4 5 triol"[All Fields] OR "sotagliflozin"[All Fields]) OR ("empagliflozin"[Supplementary Concept] OR "empagliflozin"[All Fields]) OR ("canagliflozin"[MeSH Terms] OR "canagliflozin"[All Fields]) OR ("2 3 4 ethoxybenzyl 4 chlorophenyl 6 hydroxymethyltetrahydro 2h pyran 3 4 5 triol"[Supplementary Concept] OR "2 3 4 ethoxybenzyl 4 chlorophenyl 6 hydroxymethyltetrahydro 2h pyran 3 4 5 triol"[All Fields] OR "dapagliflozin"[All Fields]) OR ("ertugliflozin"[Supplementary Concept] OR "ertugliflozin"[All Fields]) OR ("1 5 anhydro 1 5 4 ethoxybenzyl 2 methoxy 4 methylphenyl 1 thioglucitol"[Supplementary Concept] OR "1 5 anhydro 1 5 4 ethoxybenzyl 2 methoxy 4 methylphenyl 1 thioglucitol"[All Fields] OR "luseogliflozin"[All Fields]) OR ("ipragliflozin"[Supplementary Concept] OR "ipragliflozin"[All Fields]) OR "remogliflozin"[All Fields] OR ("sergliflozin"[Supplementary Concept] OR "sergliflozin"[All Fields])) AND ("heart failure"[MeSH Terms] OR ("heart"[All Fields] AND "failure"[All Fields]) OR "heart failure"[All Fields] OR ("heart failure"[MeSH Terms] OR ("heart"[All Fields] AND "failure"[All Fields]) OR "heart failure"[All Fields] OR ("cardiac"[All Fields] AND "failure"[All Fields]) OR "cardiac failure"[All Fields]) OR ("congest heart fail"[Journal] OR "chf"[All Fields])) | 927 |
| Scopus | ( ( TITLE-ABS-KEY ( sodium-glucose AND co-transporter AND inhibitor ) OR TITLE-ABS-KEY ( sglt2 AND inhibitor ) OR TITLE-ABS-KEY ( sglt-2 AND inhibitor ) OR TITLE-ABS-KEY ( sglt 2 inhibitor ) OR TITLE-ABS-KEY ( tofogliflozin ) OR TITLE-ABS-KEY ( empagliflozin ) OR TITLE-ABS-KEY ( sotagliflozin ) OR TITLE-ABS-KEY ( canagliflozin ) OR TITLE-ABS-KEY ( dapagliflozin ) OR TITLE-ABS-KEY ( ertugliflozin ) OR TITLE-ABS-KEY ( luseogliflozin ) OR TITLE-ABS-KEY ( ipragliflozin ) OR TITLE-ABS-KEY ( remogliflozin ) OR TITLE-ABS-KEY ( srgliflozin ) ) ) AND ( ( TITLE-ABS-KEY ( heart AND failure ) OR TITLE-ABS-KEY ( cardiac AND failure ) OR TITLE-ABS-KEY ( chf ) ) ) | 1725 |
| Cochrane CENTRAL | (sodium-glucose co-transporter inhibitor OR SGLT2 inhibitor OR SGLT-2 inhibitor OR SGLT 2 inhibitor OR tofogliflozin OR sotagliflozin OR empagliflozin OR canagliflozin OR dapagliflozin OR ertugliflozin OR luseogliflozin OR ipragliflozin OR remogliflozin OR sergliflozin) AND (heart failure OR cardiac failure OR CHF) | 473 |

**Table S2:** Quality Assessment of included trials

| Trial | Sequence generation | Allocation concealment | Blinding | Detection bias | Attrition bias | Other bias |
| --- | --- | --- | --- | --- | --- | --- |
| EMPA-REG OUTCOME | Low | Low | Low | Low | Low | Low |
| CANVAS | Low | Low | Low | Low | Low | Low |
| DECLARE–TIMI 58 | Low | Low | Low | Low | Low | Low |
| DAPA-HF | Low | Low | Low | Low | Low | Low |
| EMPEROR-Reduced | Low | Low | Low | Low | Low | Low |
| VERTIS | Low | Low | Low | Low | Low | Low |
| SOLOIST-WHF | Low | Low | Low | Low | Low | Unclear |

EMPA-REG OUTCOME = Empagliflozin Cardiovascular Outcome Event Trial in Type 2 diabetes Mellitus Patients; CANVAS = The Canagliflozin Cardiovascular Assessment Study; DECLARE- TIMI 58 = The Dapagliflozin Effect on Cardiovascular Events–Thrombolysis in Myocardial Infarction 58 trial; DAPA-HF = Dapagliflozin and Prevention of Adverse Outcomes in Heart Failure; EMPEROR – Reduced = EMPagliflozin outcomE tRial in Patients With chrOnic heaRt Failure With Reduced Ejection Fraction; VERTIS = Ertugliflozin Efficacy and Safety Cardiovascular Outcomes Trial; SOLOIST-WHF = Effect of Sotagliflozin on Cardiovascular Events in Patients With Type 2 Diabetes Post Worsening Heart Failure.

**Figure S1:** PRISMA flow chart


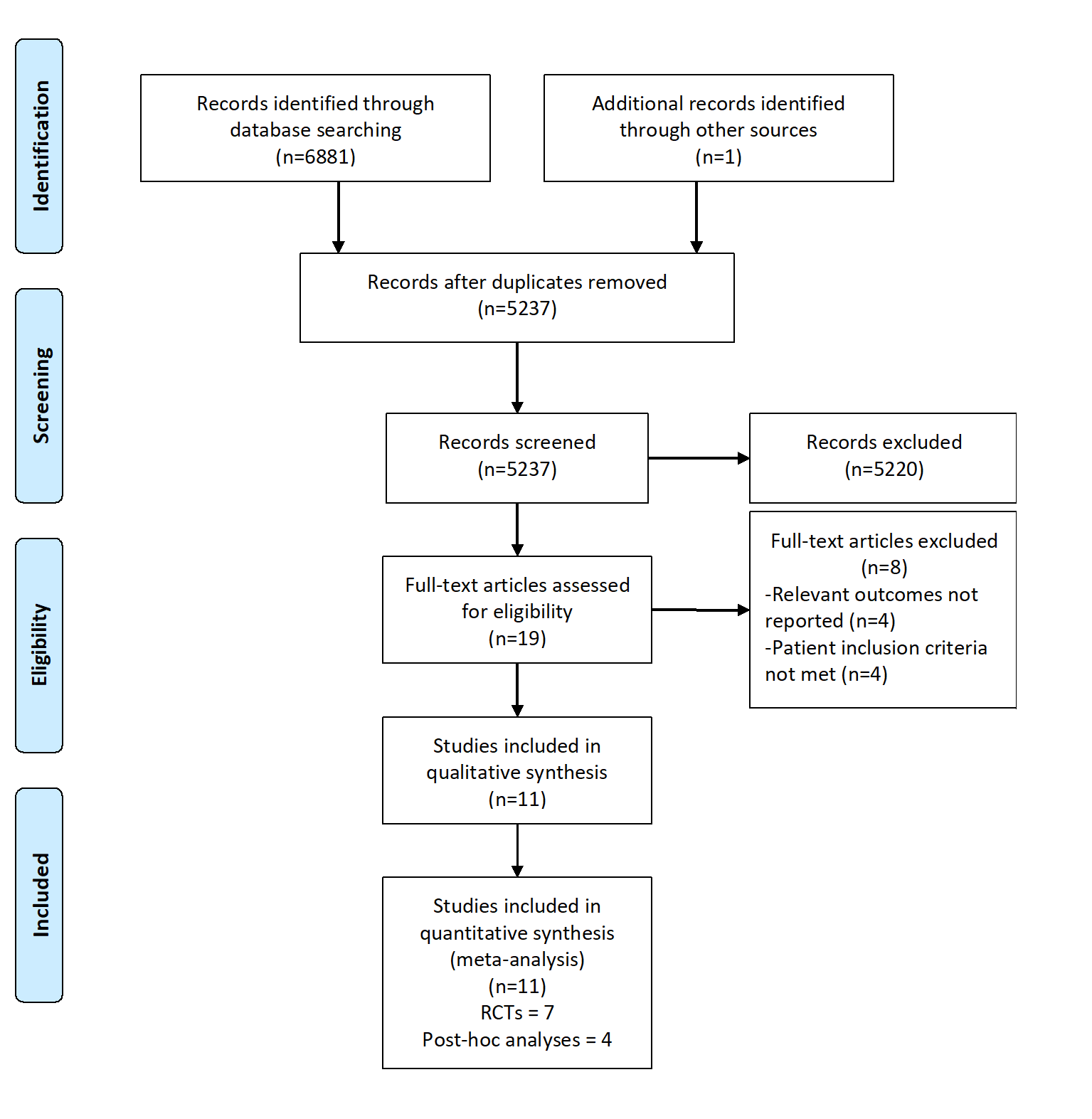


**Figure S2:** Forest plots displaying subgroup analysis according to DM status in the overall cohort of HF patients for the following outcomes: (A) composite of first HFH or cardiovascular death*; (B) total (first and recurrent) HFH or cardiovascular death; (C) First HFH; (D) cardiovascular death; (E) all-cause death; (F) Renal composite

*For subgroup analysis, the DAPA-HF trial reported a composite of first HFH, cardiovascular death and urgent visits due to worsening HF. However, urgent visits were few in this trial, and their inclusion did not significantly impact the overall effect size. Thus, we assumed that the inclusion of urgent visits would not significantly impact the effect seen in each subgroup

**(A) Composite of first HFH or cardiovascular death**

**
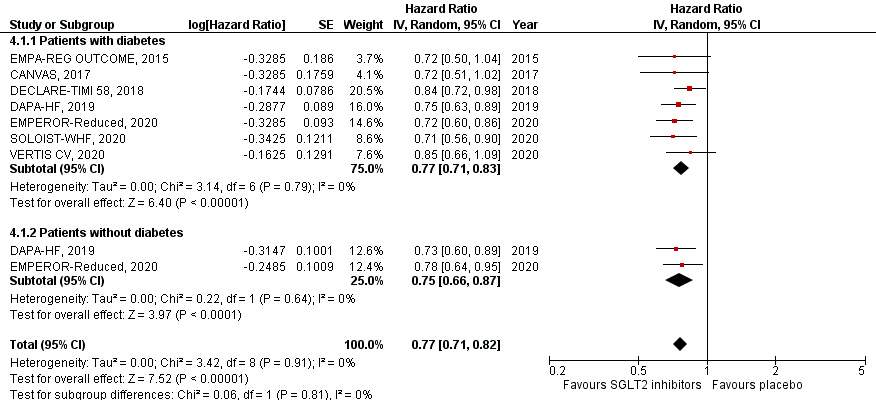
**

**(B) Total (first and recurrent) HFH or cardiovascular death**

**
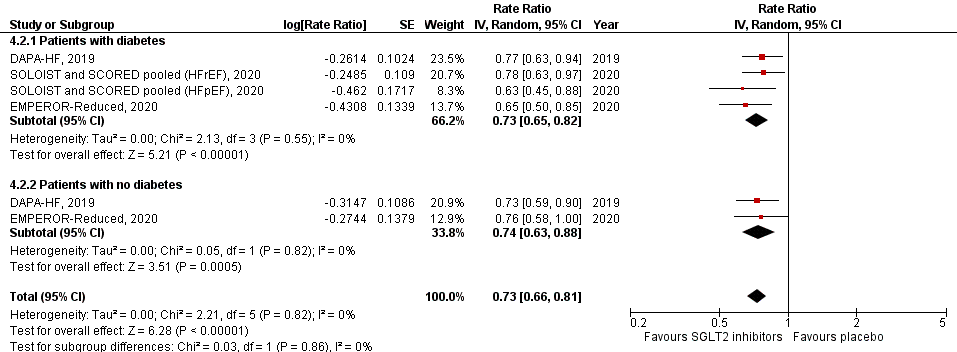
**

**(C**) **First HFH**

**
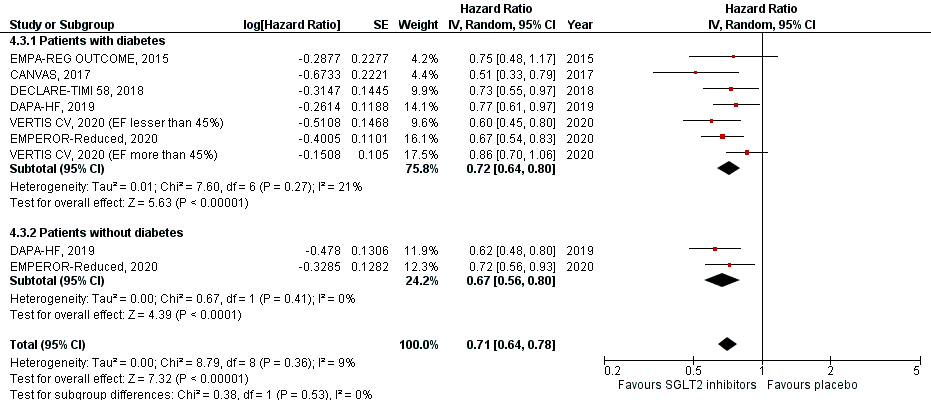
**

**(D**) **Cardiovascular death**

**
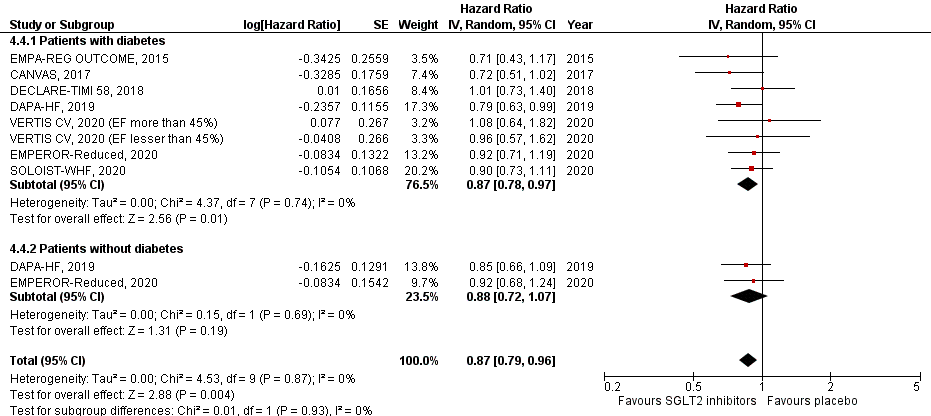
**

**(E) All-cause death**

**
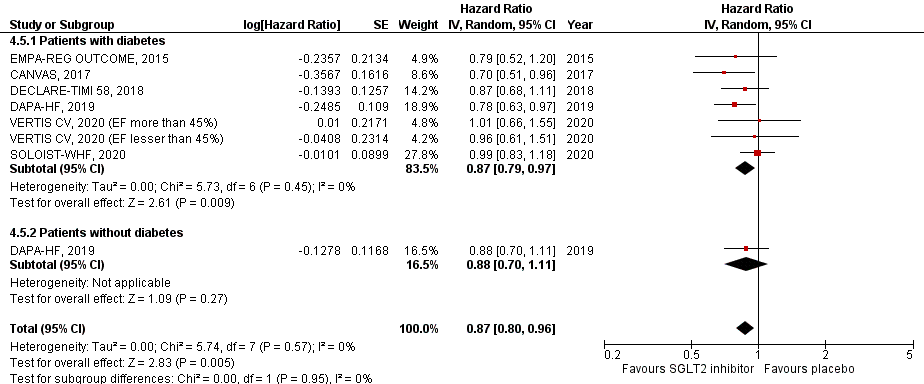
**

**Figure S3:** Forest plots displaying subgroup analysis according to DM status in the cohort of HFrEF patients for the following outcomes: (A) composite of first HFH or cardiovascular death*; (B) total (first and recurrent) HFH or cardiovascular death; (C) First HFH; (D) cardiovascular death; (E) all-cause death; (F) Renal composite

*For subgroup analysis, the DAPA-HF trial reported a composite of first HFH, cardiovascular death and urgent visits due to worsening HF. However, urgent visits were few in this trial, and their inclusion did not significantly impact the overall effect size. Thus, we assumed that the inclusion of urgent visits would not significantly impact the effect seen in each subgroup.

**(A) Composite of first HFH or cardiovascular death**

**
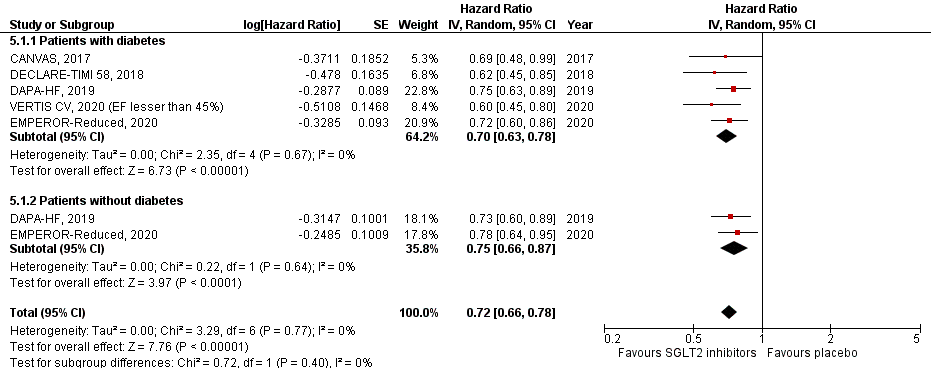
**

**(B) Total (first and recurrent) HFH or cardiovascular death**

**
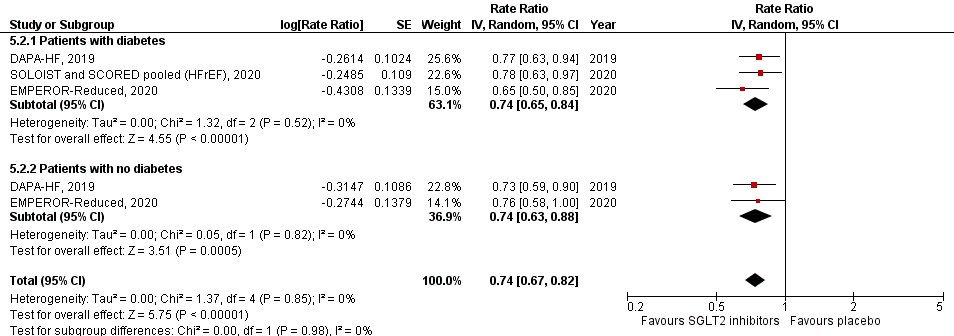
**

**(C) First HFH**

**
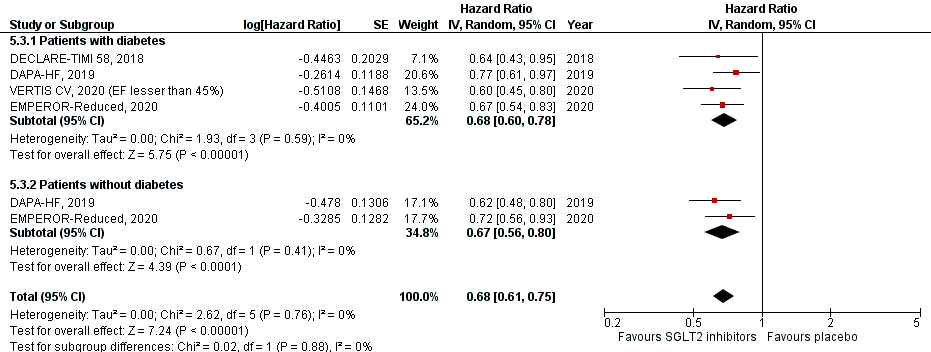
**

**(D) Cardiovascular death**

**
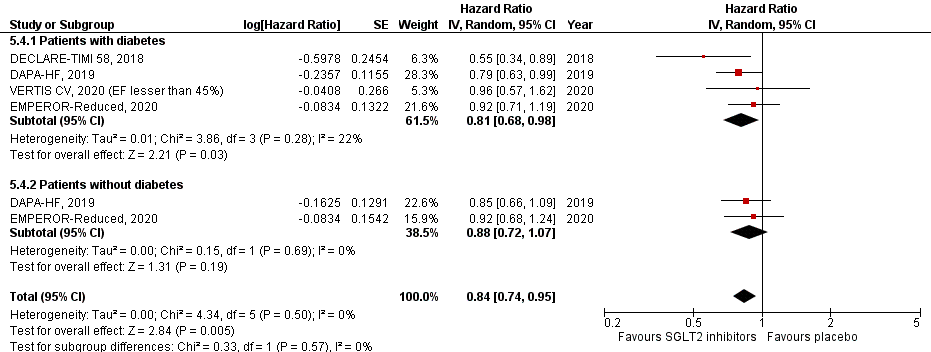
**

**(E) All-cause mortality**

**
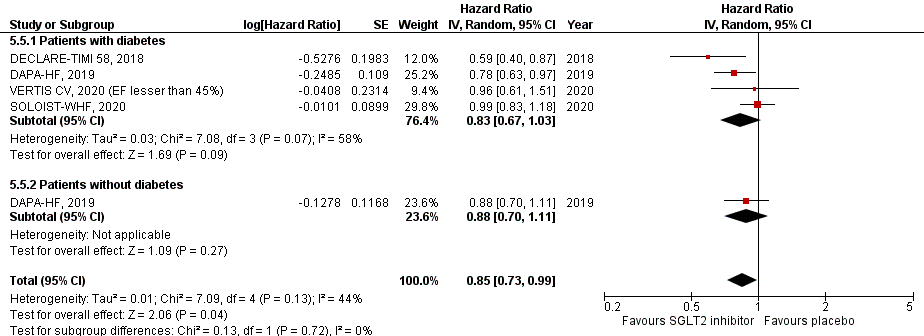
**
